# C-852-02. Demographic and Clinical Predictors of Skin Graft Loss in Burn Injuries: A National Database Study

**DOI:** 10.1093/jbcr/irag033.062

**Published:** 2026-04-06

**Authors:** Mare G Kaulakis, Catherine K Perez, Hilary Y Liu, Sarah M Tepe, Christopher J Fedor, Alain C Corcos, Jenny A Ziembicki, Francesco M Egro, Abbas M Hassan

**Affiliations:** University of Pittsburg Medical Center Mercy Burn Center, Pittsburgh, PA; University of Pittsburg Medical Center Mercy Burn Center, Pittsburgh, PA; University of Pittsburg Medical Center Mercy Burn Center, Pittsburgh, PA; University of Pittsburgh Medical Center, Pittsburgh, PA; University of Pittsburg Medical Center Department of Plastic Surgery, Pittsburgh, PA; University of Pittsburgh Medical Center, Pittsburgh, PA; University of Pittsburgh Medical Center, Pittsburgh, PA; University of Pittsburgh Medical Center, Pittsburgh, PA; Indiana University, Indianapolis, IN

## Abstract

**Introduction:**

Skin grafting is often performed in patients with full or partial-thickness burns. However, skin graft loss remains a significant complication3. This study aims to identify demographic and clinical predictors associated with receiving a skin graft and experiencing graft loss.

**Methods:**

A retrospective analysis was conducted using the Burn Care Quality Platform (BCQP) from 2013 to 2022. Demographics, burn characteristics, comorbidities, and hospitalization outcomes were analyzed using paired t-tests, chi-squared tests, and multivariable logistic regression models.

**Results:**

Of 286 478 burn patients, 24 046 (8.4%) received a skin graft, and 472 (2.0%) of those experienced graft loss.

Patients who underwent skin grafting were likely to be older (39.3 vs.36.8, *p*<.001), male (68.2% vs. 66.0%, *p*<.001), have a greater TBSA (10.7% vs. 7.3%, *p*<.001), and have full-thickness injuries (91.5% vs. 79.6%, *p*<.001). Multivariable logistic regression showed independent predictors included increasing age (OR = 1.007, *p*<.001), alcohol use (OR 1.55, *p*<.001), drug use (OR 1.85, *p*<.001), and neurologic impairment (OR 5.46, *p*<.001). Obesity (OR 0.76), diabetes (OR 0.87), and heart failure (OR 0.54) were associated with lower odds (*p*<.001).

Among patients who received skin grafts, those with graft loss were older (46.4 vs. 39.2, *p*<.001) and had greater TBSA burns (23.7% vs. 10.5%, *p*<.001). Graft loss was associated with more ICU days (25.8 vs. 7.2, *p*<.001), more operations (4.7 vs. 1.8, *p*<.001), higher in-hospital mortality (5.1% vs. 2.4%), and greater likelihood of discharge to another facility (43.8% vs. 13.6%).

Multivariable logistic regression showed independent predictors of graft loss included alcohol use (OR = 2.02, *p*<.001), drug use (OR = 1.26, *p*=.034), obesity (OR = 1.51, *p*<.001), diabetes (OR = 1.30, *p*=.001), hypertension (OR = 1.27, *p*=.001), and neurologic impairment (OR = 1.68, *p*=.031). Larger TBSA (OR = 1.02), trunk burns (OR = 2.33), and lower extremity burns (OR = 2.21) were associated with increased risk (*p*<.001).

**Conclusions:**

Early recognition of these factors may help inform preventative strategies, perioperative planning, and postoperative care to improve patient outcomes and reduce graft failure rates.

**Applicability of Research to Practice:**

Highlighting key risk factors for graft loss can inform perioperative planning, patient counseling, and postoperative care. Incorporating risk stratification into routine practice may help optimize resource use and reduce graft failure rates.

**Funding for the study:**

N/A.

